# Some personal reflections relating to psychotherapy

**DOI:** 10.4103/0019-5545.44756

**Published:** 2008

**Authors:** C. Shamasundar

**Affiliations:** 250, 43^rd^ Cross, 9^th^ Main, 5^th^ Block, Jayanagar, Bangalore 560 041, India

**Keywords:** Assumptive-world, psychotherapy, desirable therapist qualities, mental health, coping skills, values and attitudes

## Abstract

This article briefly sketches five important beliefs that shaped the evolution of my professional assumptive-world in respect of psychotherapeutic practice. They are: (a) Every human has some knowledge of what we call psychology and sociology. A professional too has a share of this, which must be integrated with his or her professional knowledge. (b) Therapist's professional knowledge should ideally be practised in his or her personal life. (c) Ideal qualities, which promote mental health are similar to desirable therapist qualities. (d) Mental health is effective management of one's miseries and illnesses. (e) Coping skills are behavioural expressions of ideal values and attitudes.

## ASSUMPTIVE WORLD CHANGES WITH TIME

This is a brief narrative about how my professional assumptive-world relating to psychotherapy has changed over three decades. Of course, there have been numerous other changes not directly related to this topic. I am using the word ‘assumptive-world’ to mean an individual's understanding of himself and his environs in a fairly cohesive, internally consistent, and integrated manner. Naturally, this assumptive-world will not be static, nor will it be perfect in details. The internal consistency will always be at the cost of ignoring uncomfortable details and the process of personal evolution involves an on-going struggle to master these details. I hope it will make interesting reading, and in the bargain, offer a few themes or issues for fellow professionals to reflect upon.

One of the common recurring elements in these changes was the issue of intellectual-knowledge versus living-knowledge. As this issue has numerous ramifications, it will be elaborated further in different though related contexts and thus, may repeat itself a few times.

## KNOWLEDGE OF PSYCHOLOGY IS UNIVERSAL

My first exposure to psychological literature was when, as a medical student, I read a popular book on psychology by Freud (Psychopathology of Everyday Life). What he had to say seemed so starkly real, so much a part of my own being, that I was surprised that psychology was so easy to learn.

After studying psychiatry, I was convinced that in order to function in a social system, all humans have to be psychologists and sociologists to some degree or the other. They differ from the academics only in respect to less extensive (quantitatively less) and less precise (qualitative) knowledge, a higher number of myths, and the absence of sophisticated terminology. In this context, it is necessary to remind overselves that even (we) the academicians are not free from myths! For example: (i) Relative consistency of personality as opposed to the ability to change as a supreme human quality, (ii) Subjective-objective dichotomy as opposed to the established psychological principle that perception, cognition, etc. are subject to bias, dependent on such personality factors as values, attitudes, beliefs, prejudices.

However crude his knowledge of psychology may be, the common man is not aware of the fact because that knowledge is a part of his being, his day to day life. In our own right as members of a society, we are already lay psychologists. In addition, as part of professional training, we formally learn high-brow, academic psychology. Thus, we mental health professionals are subject to a dichotomy, a sophisticated intellectual knowledge and a simple living-knowledge. The problem is whether the lay and the professional knowledge remain isolated or get integrated. If we are not careful, we may find ourselves riding in two boats, the lay and the professional, one leg in each! Such a dichotomy is a potential source of stress because of the dissonance between the ideal-self and the working-self and a danger to that individual's mental health. The question is, to what extent do we actively nurture an integration of this knowledge so that the evolving whole keeps becoming part of our being? This realization was the first turning point in my professional assumptive-world.

### Relevance of the universality of psychological knowledge to practice of psychotherapy

Ideally, the psychotherapeutic management of a client should be relatively easy, as he (or she) already has a ready, living-knowledge of psychology that he (or she) uses in day-to-day life. All that needs to be done is upgrading, correcting, and refining of the client's own lay knowledge. But, the language of the therapeutic procedure must be one that the client's assumptive world is familiar with. Here, I am using the word language not in the sense of English, Hindi, or Telegu, but in the sense of idioms, concepts, proverbs, etc.

## TO KNOW IS TO BECOME

An Indian folk-tale that beautifully demonstrates the above distinction between intellectual and living knowledge has been narrated in one of my earlier articles (i).The ideal of knowledge is ‘living’ that knowledge.

One corollary of the foregoing points can be stated thus: a mental health professional's knowledge of psychology and related disciplines, to the extent that they deal directly with the problems of life, should ideally be part of his being. This ideal may not always manifest fully, but a sincere effort must necessarily be a continuous, ongoing process. This is because of the simple, common-sense principle that one cannot give what one does not have. Again, this is because the professional's instrument (the mind) that contributes to effective management of his client's problems is the same as that which manages the problems of his (or her) own life. This fact is irrespective of the need for the professional to maintain a nominal, identifiable boundary between the self and the client in a therapeutic relationship. An aspect of this corollary is as follows: a very effective and easy way of learning psychology is by a process of continued application. By application I mean, verifying the tenets in one's own life and the lives around oneself. This verification is mostly retrospective: a past event supplying proof for a heard, read, or postulated concept. It is occasionally prospective: we would have come across a concept, and some event at a future date reminds us of that concept. Such a verification yields over a period of time a very genuine, personalised and hence, authentic knowledge of psychology and sociology, and I believe this process is the basis of the emergence of different schools of psychology.

Another corollary is the issue of therapist's genuineness, correspondence, or coherence between one's ideal-self and working-self. The ancient Indian equivalent of this is *manasa-kaya-vaacha*: coherence between feelings, thinking, speech, and action. Interestingly, genuineness (absence of pretence) is one of the desirable qualities of a therapist, and these qualities contribute to comparable therapeutic benefit by different methods of psychotherapy, as well as to the so-called placebo-effect. This realization was the second turning point in my professional assumptive-world.

### Relevance of the personalization of psycho-social knowledge to psychotherapy

Of course, a therapist's professional knowledge will be personally unique in accordance with his personality (values, attitudes, perception, cognition, etc.), but it will be authentic, which a client will naturally sense and trust.

### Desirable therapist qualities

I have described in my article on empathy[2] how the attitudes and feelings of therapists do get empathically transferred to the client and vice versa. This is the basis of therapeutic benefit to the client and professional stress to the therapist. How therapist qualities can contribute to a positive therapeutic outcome is briefly described in that paper.

The third turning point in my professional assumptive-world was when I gradually realized that desirable therapist qualities were either the same as or analogous to: 1) components of ideal human behavior as described in ancient Indian literature and 2) attitudinal, behavioral, and personality qualities that correlate with well-being. I have shown this fact in the form of a table in an article of mine,[3] and have reproduced that table as Table 1 here. One of the implications of this fact is that an ideal person is more likely to be mentally healthy and at the same time have psychotherapeutic potential, irrespective of whether or not he (or she) is aware of the fact.

**Table 1:** Comparing desirable therapist qualities with components of ideal human behavior and factors that correlate with well-being (mental health)

Therapist qualities	Ideal behavior	Well-being
Hopefulness		Hopefulness
Self-confidence	Self-confidence	Confidence
	Courage	Fearlessness
Honesty	Honesty	
Integrity	Sincerity	Sincere sense of responsibility and duty
	Truthfulness	
Empathic	Affectionate compassionate	Affectionate
Unconditional positive regard	Respect and goodwill for others	Response to positive qualities
Non-judgmental tolerance and acceptance		Tolerance
Acknowledgement of the client's rights		
Genuineness	Genuineness	
Ability to monitor one's own behavior	Not allowing one's own behavior to be influenced by anger, hatred, fear, or pride	Not allowing personal reasons to interfere with good conduct

Notes: In the above table, an attempt is made to group together approximately similar components. The few empty cells mean that the literature that was scanned did not contain corresponding information. But, we can fill them up from our experience.

Another implication of the above fact is that we the professionals have to struggle to be healthy and to learn effective psychotherapeutic skills because, by current social compulsions, we have deviated from the ideal. A very easily understandable example of such a deviation is the degree of pretence that has crept into our day-to-day life at every level and area of functioning. For example, even in the professional area, salesmanship is equated with expertise, extempore fluent verbosity with knowledge, dramatics with sincerity, and cunning or histrionic manipulativeness with administrative skills. Consequently, whether we are aware of it or not, in some therapeutic context or the other, our pretence shows through to the client. Or, our hard struggle to be genuine during the therapeutic interactions leaves us exhausted making us more vulnerable to professional stress. These are a few among numerous reasons why professionals so easily prefer to prescribe a pill rather than effectively interact; and of course, a busy practice offers a legitimate excuse.

The relevance of the desirable therapist qualities to a psychotherapist is self-explanatory. As a result of our varying degrees of deviance from the ideal qualities, we necessarily have to cultivate them with active effort.

### Mental health

A logical consequence of the foregoing points is that a professional's personal evolution should involve an integration of all aspects of one's personal and professional functioning. Such an integration constitutes the basis of his or her own mental health. Here, I am using the phrase mental health in the sense of the ability to manage the demands and stress of personal and professional functioning, as I have described in the earlier write-up.[3] This description, based on tenets from the ancient Indian scriptures is operational, and I quote: “The individual attends to all legitimate affairs of one's life in social, familial, personal and occupational areas in order to fulfill one's own and family's spiritual, affectional and material needs according to role functions, abilities, circumstances, and within the limits of righteousness (social-moral code), with attitude of hope, confidence, sincere sense of responsibility and contentment.”

In the above description, righteousness means what is good to most in the long run (Note: here, the word most is my own; in the original scriptures, it is ‘God's Creation’). Contentment does not mean a passive, masochistic suffering! It is an ability to courageously accept a cross-sectional situation (circumstances) with dignity and composure without pathological responses. At the same time, the contented individual is expected to plan a future course of action and execute it with hope and confidence, ready to face the consequences in a contented manner.

Another aspect of this description of health is related to what the ancient Indian wisdom says about human life: “In human life, miseries and illnesses are inevitable,” a fact, none of us can deny. A few inevitable logical consequences of this fact constituted the fourth turning point in my professional assumptive-world. They are: 1) The state of mental health is not dependent on the presence or absence of inevitable miseries (difficulties) and illnesses. 2) Therefore, it must be definable by how one manages one's miseries and illnesses. 3) Thus, it (its presence or absence) becomes evident only when life is challenged by difficulties, and not when everything is happening smoothly according to one's expectation. 4) Skills in management of miseries and illnesses are nothing but coping skills, which include components of ideal behavior, like emotional maturity (ability to tolerate unpleasantness in oneself and others, and this also includes hardiness), good-will for fellow beings, awareness of one's abilities and limitations, reality orientation, courage to learn by trial and error (ability to take calculated risks and responsibility to face the consequences). 5) A social system that is over-protective towards its children without allowing them opportunities for trial and error-learning, or an educational system that is heavily weighted towards academic learning at the cost of social skills will breed a generation with deficient coping skills. These postulates may become measurably evident after a few generations, if the current social trends continue.

The relevance of the above concept of health for the psychotherapist is for him (or her): 1) not to become a part of the client's unrealistic, utopian concept of well-being as freedom from symptoms or difficulties in life and 2) not to deprive the client of one's own responsibility for one's well-being. A general tendency in clinical practice is to unwittingly reinforce a client's attitude or sense of non responsibility (for one's own state of affairs) by ascribing his (or her) illness, behavior, or even crimes to genes, chemicals, and environment. Such an attitude is further amplified by heavy dependence on physical and chemical methods of management. Thus, the client loses the opportunity to learn to become responsible for one's own health or illness. In this context, it is interesting to note that many effective management techniques, even in areas such as geriatric and child psychiatry, depend on the principles of learning, which requires the client's cooperation.

### The relation of coping skills to values and attitudes

If we reflect on what these coping skills really are, sooner or later, the chain of reasoning will lead us to a thesis that most of them, if not all of them, are the behavioral end-products of values and attitudes that are culturally cherished. Also, as mentioned earlier, these values and attitudes are analogous to desirable therapist qualities. This realization was the fifth turning point in my professional assumptive world.

Deficiency in coping skills due to corresponding deviance in values and attitudes leads to converting routine events in life to severe stress by a process of pathological perception. This stress leads to severe conflicts, which in turn elicit exaggerated responses. Sooner or later, a vicious cycle gets established, which generates severe psycho-somatic stress and an increasing dissonance between the idea-self and the working-self. This dissonance itself correlates with psychological morbidity. The consequences will be an emergence or manifestation of psycho-somatic, psycho-social, or psychiatric symptoms or syndromes. The following are a few examples:

1. Greed leads to such objectives as over-ambitiousness, not wanting to sacrifice, violation of fair means, and aggressive competitiveness. For example: (a) Over-ambitiousness will be blind to reality and disregards the limitations of one's own abilities, prevailing circumstances, and social responsibilities. (b) Not wanting to sacrifice needs little explanation. After all, one of the essential principles in the decision-making process in life is exercising a choice (What advantage am I prepared to sacrifice for what other advantage? What disadvantage am I prepared to tolerate in order to avoid what other disadvantage, etc.?). (c) Expecting quick results, without the price of hard work by the use of exploitative, manipulative, and dishonest social interactions in place of co-operative good-will with fairness.
2. Emotional immaturity and low frustration tolerance are self-explanatory.
3. Lack of courage to: (a) be flexible; (b) experiment with alternative strategies, and (c) face possible adverse effects with readiness to manage them as best as one can.
4. Pretence (absence of genuineness), which is associated with incongruity between: (a) feeling, thinking, speech and action, that is, between different components of one's psyche, which constitutes dissociative pathology; (b) one's ideal-self and working-self, which correlates with psychological morbidity as mentioned earlier, and (c) reality and one's assumptive-world, which augments potential stress.

It has to be noted that these above factors do not negate the validity and reliability of existing theories of psycho-pathology, but, can be considered as complementary addendums.

The relevance of these above issues in psychotherapy is: 1) The client has to be guided to realize his own responsibility for what he is and for what he wants to become. 2) The client will benefit from therapy to the extent that the therapeutic insights become his (or her) own living-knowledge and not merely intellectual knowledge (this is the reason why many of our educated clients suffer despite knowing all the psycho-social explanations).

### There is nothing new under the sun

There is nothing new or novel about these above ideas that I have presented. They have existed in the past. The majority of, if not all, fellow professionals know them. They can be found scattered in our current professional literature, too. The younger generation of mental health professionals will experience them at different stages of their professional life. I have just clubbed them together. I believe that these issues deserve more elaborate thinking and research in future.
